# COVID‐19 related lung pathology: old patterns in new clothing?

**DOI:** 10.1111/his.14162

**Published:** 2020-08-04

**Authors:** Andrew G Nicholson, Michael Osborn, Anand Devaraj, Athol U Wells

**Affiliations:** ^1^ Department of Histopathology Royal Brompton and Harefield NHS Foundation Trust and National Heart and Lung Institute Imperial College London UK; ^2^ Department of Cellular Pathology Northwest London Pathology Imperial College London NHS Trust and Mortuary Lead Nightingale NHS Hospital London UK; ^3^ Department of Radiology Royal Brompton and Harefield NHS Foundation Trust London UK; ^4^ Interstitial Lung Disease Unit Royal Brompton and Harefield NHS Foundation Trust and National Heart and Lung Institute Imperial College London UK

**Keywords:** COVID‐19, interstitial pneumonia, lung, pathology

As the wave of COVID‐19 disease has spread across the globe, so medical publications have followed as part of scientific efforts to characterise, manage and hopefully prevent this disease. Papers pertaining to pathology have, by their very nature, lagged slightly behind those describing clinical and imaging cohorts, but we are now seeing increasing numbers of publications that give us insight into how human tissues are infected and damaged by the virus, three in this particular issue of *Histopathology*.

Menter *et al*. report a cohort of 21 patients dying of COVID‐19 infection. Acute lung injury was the main pathology at autopsy, primarily showing the exudative phase of diffuse alveolar damage (DAD), with a minority displaying the organising (or proliferative) phase.^1^ In addition, there was frequent co‐existent vascular pathology, mainly thrombotic and more rarely vasculitic. In earlier small series and single cases, DAD was observed both with and without vascular damage.^2^, ^3^, ^4^ Larger series provide a broader picture of the spectrum of COVID‐related lung pathology,^1^, ^5^, ^6^ as well as confirming associations with male gender, hypertension, obesity and finding unexpected comorbidities such as senile cardiac amyloidosis.^1^

The identification of vascular lung pathology in some, but not all, patients is also consistent with clinical and imaging data in relation to pro‐thrombotic states,^7^, ^8^ and we can conceptualise that this breadth of pathology findings represents several pathways through which patients, especially those who suffer severe disease, progress to a final common outcome of DAD. These include direct damage to the lungs by the virus and secondary bacterial infections, and systemic effects that manifest through immune dysregulation and increased levels of thrombosis (Figure 1). Indeed, it is likely that these pathways frequently interplay, and the next steps for the scientific community are unravelling these within individuals at the earliest stage possible to guide management.

**Figure 1:**
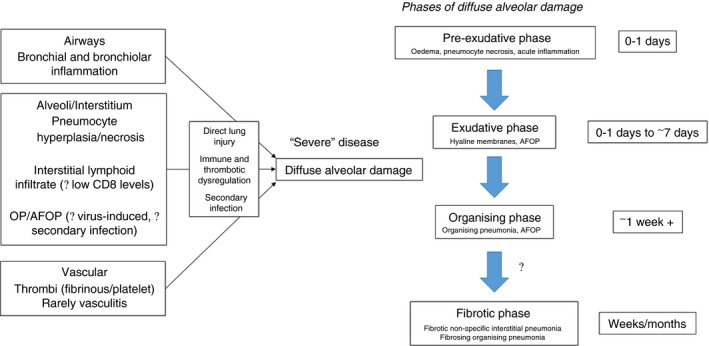
COVID‐19 virus‐related lung histopathological features. Histological features and patterns may vary and coexist dependent upon (**A**) the time‐point in disease evolution, (**B**) severity of disease and (**C**) the pathway(s) induced by viral infection.

In the second paper, Zeng *et al*. describe a patient operated on for a benign lung lesion before developing symptoms of COVID 19 infection.^9^ Reported cases of pre‐mortem pathology are rare, and this case is a snapshot of what appears to be the disease in the earliest microscopically visible phase of DAD/acute lung injury. Although no hyaline membranes are seen there are oedematous exudates, one of the earliest features of DAD, identifiable on microscopy.^10^ Typically, hyaline membranes take hours to develop. Similar features have been reported in lung cancer resections of patients later shown to have COVID‐19 infections.^11^, ^12^

The paper by Zeng *et al*. is also interesting for its description of a moderately intense lymphoid infiltrate within the interstitium, focally cuffing the pulmonary vasculature. This is also seen in other reports,^2^, ^4^, ^13^ and is in keeping with some patients at autopsy reported by Menter *et al*., whereas a relative dearth of lymphoid infiltrate is reported in other cases.^1^ Zeng *et al*. report a relative lack of CD8^+^ lymphocytes in the alveolar interstitium, also reported in the haematological literature.^14^ Others report CD8^+^ lymphocytes outnumbering CD4^+^ lymphocytes.^2^ Plasma cells and CD4^+^ lymphocytes were, however, present in the paper by Zeng *et al*., the latter again reported by others in bronchoalveolar lavage^15^ and post‐mortem biopsy, respectively.^4^ At present these are only very small series, but clearly this micro‐environment of interstitial inflammation and its relationship to the systemic, often lymphopaenic,^16^, ^17^ inflammatory state is a key subject for future investigation as we move beyond basic morphology in attempting to understand the pathogenesis of this disease and the interplay between pathways.

As data amass on COVID‐19‐related lung pathology, it is worth taking a step back and reviewing the variety of patterns of interstitial lung disease that have been reported since the beginning of the pandemic, ranging from interstitial lymphoid infiltrates, oedematous exudates, acute fibrinous and organising pneumonia (AFOP)^18^, ^19^ and DAD in both the exudative and organising phases. Their ‘uniqueness’ has often been the thrust behind publication, but we believe that what one is seeing is the above pathways being ‘freeze‐framed’ and variably sampled at different time‐points in their evolution, additionally influenced by both extent of insult and individual patient susceptibility, be that genetic or acquired in the form of comorbidities (Figure 1). DAD is one of the histological patterns of interstitial pneumonia published in the ATS/ERS consensus document in 2002,^20^ reviewed again in 2013.^21^ This classification unified the clinical management of patients with interstitial lung disease across disciplines, a field that had hitherto been muddied by excessive terminology interpreted in different ways. AFOP (predominantly fibrinoid material forming intra‐alveolar buds) is also accepted as part of the classification system as a histological pattern that probably reflects a rate of response to lung injury between that of organising pneumonia (buds of granulation tissue) and exudative DAD [linear fibrinoid material (hyaline membranes) lining alveoli]: in essence, somewhere between subacute and acute response to lung injury.^21^ First reported by Beasley *et al*. in 2002,^22^ it is an accepted histological pattern of interstitial pneumonia, but is viewed neither as an idiopathic clinicopathological entity nor a disease‐specific histological pattern, as there are a variety of causes (drug, infections) and associations (connective tissue disorders, post‐transplantation).^22^, ^23^, ^24^, ^25^, ^26^, ^27^, ^28^, ^29^, ^30^, ^31^, ^32^, ^33^ It is also not infrequent to see areas of OP, AFOP and DAD in the same autopsy from a patient dying of acute lung injury, and computerised tomography data describe some patients with COVID‐19 who have disproportionate consolidation prior to, or alongside, classic DAD,^34^, ^35^ which may reflect a cohort with OP/AFOP as a sequelae of immune dysregulation, as seen in anti‐synthetase syndrome.^36^ Therefore, the presence of AFOP may reflect the final common pathway of DAD, systemic virally induced immune dysregulation,^37^ secondary infection or any combination (Figure 1).

Therefore, it is important that a balance is maintained between splitting COVID‐19 into a multitude of histological patterns for research into pathogenesis and merging them all together as ‘COVID‐lung’ for the purposes of management, as is the case with other interstitial lung diseases.^38^ Unravelling this interplay between pathways may well be the middle‐ground for smarter splitting in terms of pathogenesis, and smarter merging in terms of targeting treatment to block specific pathways. Finally, this overlap with interstitial lung disease may well prove increasingly important as we start to see the chronic sequelae of COVID‐19 infection, given that the interstitial lung disease community has long been aware that fibrotic non‐specific interstitial pneumonia and organising pneumonia progressing to fibrosis are not infrequently seen in survivors of DAD/acute lung injury. As yet, we have little idea of the extent of chronic pathology in survivors, let alone those with milder infections not requiring hospitalisation.

The COVID‐19 pandemic also has seen many indirect consequences, as shown in the third paper by Binder *et al*., reporting an individual suffered erosive gastrointestinal injury following intentional repetitive sublethal ingestion of ethanol‐based hand cleaner.^39^ Notwithstanding the musings of certain world leaders, this highlights the wide‐reaching effects of this disease, both physical and psychological. The risks to health workers should also not be underestimated, given that five individuals involved in treating the one patient later tested positive in the paper by Zeng *et al*.^9^ It is impossible to say how extensive the ramifications of this chapter in medical history will be. As these three papers highlight, this is not just in terms of accrual of clinical data to combat the disease. We are seeing resultant and rapid changes in our work practices, the need for social distancing meaning that many support services have jumped to the front of the information technology queue. As an example, the paper from Menter *et al*. has wonderful direct links to whole slide images. Our approach to all forms of education will surely change.^40^

However, the basics still apply. The autopsy remains a valuable source of information which, whether it be a full autopsy or minimally invasive, if coupled with thorough sampling and retention of tissue for research and taken with appropriate consent, is key to understanding diseases and helping in their prevention.^41^ As with many lessons learned as a result of this pandemic, it is hoped that the value of autopsy, suddenly remembered, is not forgotten once more.

## Conflicts of interest

None.

## Author contributions

All authors contributed to the writing and editing of the manuscript.
